# High‐Temperature Driven Recrystallization for Stable Dopant‐Free α‐FAPbI_3_ Perovskite Solar Cells

**DOI:** 10.1002/advs.202408684

**Published:** 2024-11-11

**Authors:** Lingbo Xiao, Xiaoli Xu, Jie Zhao, Chen Wang, Zheng Lu, Lutao Li, Liang He, Yu Chen, Guifu Zou

**Affiliations:** ^1^ Department of Applied Physics Zhejiang University of Science and Technology Hangzhou Zhejiang 310008 P. R. China; ^2^ College of Energy Soochow Institute for Energy and Materials Innovations Jiangsu Key Laboratory of Advanced Negative Carbon Technologies and Key Laboratory of Advanced Carbon Materials and Wearable Energy Technologies of Jiangsu Province Soochow University Suzhou 215000 P. R. China; ^3^ College of Materials and Chemistry China Jiliang University Hangzhou 310018 P. R. China; ^4^ Jiangsu Key Laboratory for Science and Applications of Molecular Ferroelectrics Southeast University Nanjing 211189 P. R. China; ^5^ National Photovoltaic Engineering Research Center LDK Solar Co., Ltd Xinyu 338032 P. R. China; ^6^ Beijing Synchrotron Radiation Facility Institute of High Energy Physics Beijing 100049 P. R. China

**Keywords:** damp‐heat stability, dopant‐free, FAPbI_3_ perovskite solar cells, hot‐press, recrystallization

## Abstract

High temperatures facilitate the formation of stable, high‐crystallinity α‐FAPbI_3_ films but can lead to the volatilization of organic components in perovskites. Here, a 300 °C hot‐press‐assisted recrystallization strategy is reported to grow stable phase‐pure α‐FAPbI_3_ film without any dopants. High temperature can promote the transformation of δ‐FAPbI_3_ to α‐FAPbI_3_, and induces recrystallization to relieve strain. The applied pressure creates a confined space that effectively prevents the volatilization of organic components in perovskite. The α‐FAPbI_3_ film prepared by hot‐press at 300 °C achieves an average grain size of ≈3 µm (with grains up to 10 µm) and demonstrates excellent damp‐heat stability, showing no significant change after 20 s in deionized water. The result solar cell delivers a power conversion efficiency as high as 24.06% and retains >70% of their initial efficiency value after 1000 h at 85 °C and 85% relative humidity.

## Graphical abstract

Phase‐pure dopant‐free α‐FAPbI_3_ perovskite films are being prepared by hot‐press assisted annealing at 300 °C. Higher temperature can promote the transformation of δ‐FAPbI_3_ to α‐FAPbI_3_, induce recrystallization to relieve strain. The applied pressure creates a confined space that effectively prevents the volatilization of organic components in perovskite. The result solar cell achieves a PCE of 24.06% and demonstrates ultra‐high damp‐heat stability.

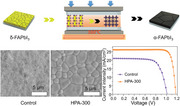

## 1 Introduction

Metal halide perovskite solar cells (PSCs) have garnered significant attention over the past decade due to their low‐cost fabrication, continuously improving power conversion efficiencies and versatility.^[^ ^1^, ^2^, ^3^ ^]^ However, poor stability is a major obstacle to the commercialization of PSCs.^[^ ^4^, ^5^ ^]^ Among metal halide perovskites, *α*‐phase formamidinium lead triiodide (*α*‐FAPbI_3_) stands out as an ideal candidate for efficient, stable perovskite solar cells due to its optimal bandgap and improved thermal stability.^[^ ^6^ ^]^ α‐FAPbI_3_ has a Goldschmidt tolerance factor (*t*) close to 1 (0.99), which is nearly a perfect cubic perovskite structure with ignored distortion theoretically (*t* is an empirical index widely used to predict the formation of different crystal structures of ABX_3_. The value of *t* can be calculated according to *t* = (r_A_+r_B_)/ (2r_B_+2r_X_)^1/2^, r_A_, r_B_, and r_X_ represent the ionic radii of A‐, B‐, and X‐site ions, respectively).^[^ ^7^ ^]^ However, photo‐inactive δ‐FAPbI_3_ is easily formed because of its low formation energy, the residual δ‐FAPbI_3_ forces α‐FAPbI_3_ to undergo a reverse phase transition back to δ‐FAPbI_3_ at room temperature, which is not conducive to the preparation of efficient and stable perovskite solar cells.^[^ ^7^, ^8^, ^9^ ^]^ To solve this problem, the most common strategy is alloying FAPbI_3_ with MA^+^, Cs^+^, and Br^−^ etc. to inhibit the involvement of δ‐FAPbI_3_, but these dopants can adversely widen the bandgaps, induce local phase separation and microscopically heterogeneous cation distribution which deteriorate the stability and efficiency of perovskite solar cells.^[^ ^10^ ^]^ Recently, researchers have been investigating various additives and dopants to develop stable and efficient α‐FAPbI_3_‐based perovskite solar cells. Additives such as isopropylammonium chloride, methylenediammonium dichloride (MDACl_2_), MASCN, and methylamineformate (MAFA) have been shown to maintain the narrow bandgap of *α*‐FAPbI_3_ and prevent phase segregation issues associated with multi‐component systems.^[^ ^11^, ^12^, ^13^ ^]^ However, strategies to prepare phase‐pure and stable *α*‐FAPbI_3_ not relying on processing additives are rarely reported.

High‐temperature annealing not only benefits the formation of stable α‐FAPbI_3_, but also improves the quality of the perovskite film.^[^ ^14^, ^15^, ^16^, ^17^ ^]^ However, traditional annealing temperatures are kept below 150 °C to prevent the volatilization of organic components in perovskites at high temperatures. Hot‐pressing technology has been used to prepare perovskite films because it can control the crystallization process and protect the perovskite against volatilization of the organic A‐site species. Thomas et al used thermal nanoimprint and hot‐pressing recrystallization process to prepare CsPbX_3_ films composed of large crystals with micrometer lateral extension, patterned MAPbI_3_ films, and MAPbBr_3_ films, and the as‐prepared perovskite films have proven to have excellent optical properties.^[^ ^18^, ^19^ ^]^ These studies have been demonstrated as preparing high‐quality perovskite films through hot‐pressing related process. However, few reports are explored how to fabricate phase‐pure and stable α‐FAPbI_3_ films by hot‐pressing. Here, the stable phase‐pure α‐FAPbI_3_ film without any dopants is achieved via a hot‐press‐assisted (HPA) recrystallization process at 300 °C. High‐temperature treatment benefits the formation of α‐FAPbI_3_ and pressure provides a confined space to effectively prevent the volatilization of FA^+^. Interestingly, high‐temperature recrystallization induces the lattice strain and the microstrain relaxation of the α‐FAPbI_3_ film to achieve the average grain size of ≈3 µm (up to 10 µm) with low defect density and exhibits excellent damp‐heat stability (even without obvious changes in deionized water for 20 s). The study shows that the FWHM (width at half maximum) of X‐ray diffraction (XRD) at ≈14^°^ reduces from 0.143^°^ to 0.058^°^ and the Urbach energy (*Eu*) reduces from 31.8 to 21.6 meV after recrystallization. The resulting solar cells deliver a 24.06% power conversion efficiency and retain >80% of their initial value after 500 h at 85 °C and 85% relative humidity.

## 2 Results and Discussion

**Figure 1:**
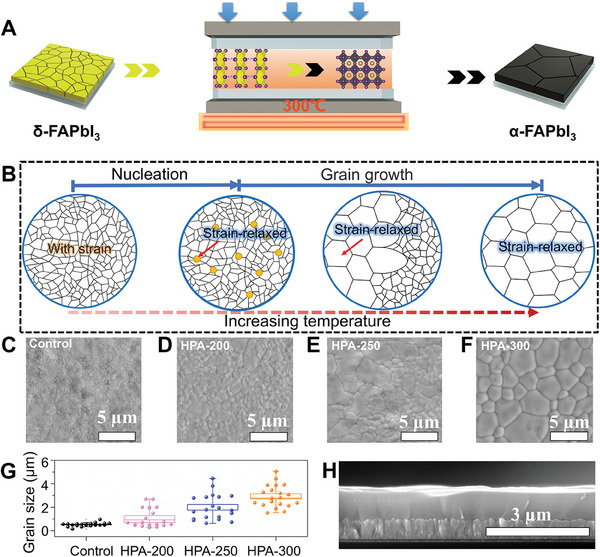
HPA process and the effect of temperature on recrystallization. A) Simplified schematic diagram of HPA process. B) Schematic diagram of recrystallization with increasing temperatures. C) Top‐view SEM image of the control FAPbI_3_ film. D–F) Top‐view SEM images of HPA‐treated FAPbI_3_ film at different temperatures 200 °C E) 250 °C F) 300 °C. G) Statistical graph of the grain size of FAPbI_3_ films under different annealing conditions H) Cross‐sectional SEM image of HPA‐300 FAPbI_3_ film.

**Figure** **1**A shows a schematic diagram of the HPA process. The δ‐FAPbI_3_ film is prepared by spin coating of a mixed solution of PbI_2_ and FAI and then preheated at 70 °C. After that, a bare FTO is covered on top of it. Finally, it is put into a hot‐press machine for HPA annealing under a pressure of 0.5 MPa. For a detailed process, please refer to Figure S1 (Supporting Information). Figure 1B illustrates the recrystallization processes accompanying HPA treated with the increase of temperature, including phase transformation from δ‐FAPbI_3_ to α‐FAPbI_3_, recrystallization nucleation, and grain growth. Traditionally, the annealing temperature of FAPbI_3_ at 150 °C can convert the most δ‐FAPbI_3_ to α‐FAPbI_3_, but there is still a small amount of δ‐FAPbI_3_ remaining in the film. Meanwhile, the FAPbI_3_ films often exhibit very small grain size which adversely affects the stability and efficiency of perovskite solar cells.^[^ ^20^ ^]^ As the temperature increases, recrystallization nucleation first occurs, as new strain‐relaxed grains tend to be generated in larger distortion regions, and then gradually consume the surrounding deformed matrix until the deformed structure is completely reorganized into new undeformed equiaxed crystals. Usually, there are more defects, dislocations, and multi‐twinning at grain boundaries than at grain interior, the energy of the grain boundary is always higher than the energy inside the grain, and these make the grain boundaries in an unstable state. When polycrystalline materials are annealed at sufficiently high temperatures, grain boundaries move and rearrange so as to increase the average grain size and decrease the grain boundary area per unit volume (*Science* 2010, 328, 1138–1141). Therefore, the driving force for grain growth is the decrease in grain boundary energy. Figure 1C shows the top‐view SEM image of the control FAPbI_3_ film (conventional annealing at 150 °C, enlarged view can be seen in Figure S2, Supporting Information), the morphology of the film shows grain size smaller than 1 µm. Figure 1D–F shows the top‐view SEM images of HPA‐treated FAPbI_3_ film (the temperature is marked after HPA, for example, HPA‐200 denotes the HPA process at 200 °C). As the increasing temperature, FAPbI_3_ undergoes the process of recrystallization nucleation and grain growth. As can be seen from Figure 1C–F, as the temperature rises and the recrystallization process proceeds, the grains of the perovskite film gradually increase, and when the temperature reaches 300 °C, a uniform α‐FAPbI_3_ film composed of large grains is formed. The original SEM images of the control and HPA‐300 FAPbI_3_ films in Figure S3 (Supporting Information) show that the grains of the FAPbI_3_ films are significantly increased after recrystallization. The effect of pressure on the morphology of perovskite films is shown in Figure S4 (Supporting Information), it can be seen that the film formed by conventional annealing at 250 °C for 10 min is composed of a large number of plate‐like structures of PbI_2_, which is due to the volatilization of the organic part in FAPbI_3_ and can be verified by the corresponding XRD in Figure S4g (Supporting Information). As the pressure increases, the volatilization of the organic part decreases significantly, A dense pore‐free α‐FAPbI_3_ film can be formed at a pressure of 0.5 MPa. This suggests that our HPA method provides a confined space to effectively prevent the volatilization of FA^+^, resulting in a good formation of α‐FAPbI_3_. Using conventional annealing at 250 °C for different times, it can be seen that perovskite decomposes easily at high temperatures (Figure S5, Supporting Information). The grain distribution statistics in Figure 1G show that the grain size of the α‐FAPbI_3_ film increases with the increase of annealing temperature and reaches the maximum value at 300 °C. The average grain size of HPA‐300 α‐FAPbI_3_ film is ≈3 µm which is much larger than the control α‐FAPbI_3_ film. The largest one can reach ≈10 µm (as shown in Figure S6, Supporting Information). In order to prove that the high‐temperature recrystallization process is not just the grain growth on the surface of FAPbI_3_ film, but the growth of the entire grain. The cross‐sectional SEM is performed and the results are shown in Figure 1H. It can be seen from the results that the entire grain of the FAPbI_3_ film has been grown, and it is in good contact with the bottom surface with fewer holes, which is more conducive to the transport of carriers in the perovskite through the bottom electrode. The wider field of view cross‐sectional SEM image of the control FAPbI_3_ film and HPA‐300 FAPbI_3_ film in Figure S7 (Supporting Information) shows that the recrystallization process induced a pinhole‐free FAPbI_3_ film.

Hot‐pressing also affects the volatilization of the solvent DMSO, thereby affecting the crystallization process of perovskite. Therefore, we explored the relationship between preheating time and perovskite film morphology, and the results are shown in Figure S8 (Supporting Information). It can be seen that the grain size of the perovskite film decreases as the preheating time increases, but too short a preheating time will cause obvious holes in the perovskite film. Through XRD and FTIR spectrum of δ‐FAPbI_3_ films with different preheating times, we found that the residual amount of DMSO affects the crystallization process of the perovskite film, thereby affecting its morphology. Especially, as the preheated time increased to 60 s,180 s, and even 60 s at 150 °C, the grain size of FAPbI_3_ films still showed significant growth compared with the reference sample (Figure S8a, Supporting Information), which indicates DMSO is not necessary for the recrystallization of perovskite films at high temperatures, but high‐quality α‐FAPbI_3_ films can be formed by properly controlling the preheating time. At the same time, we also used this method to prepare MAPbI_3_ and FA_0.9_Cs_0.1_PbI_3_ perovskite films. By adjusting the temperature, the morphology of the film was effectively improved, indicating that this method has a certain universality (Figure S9, Supporting Information).

**Figure 2:**
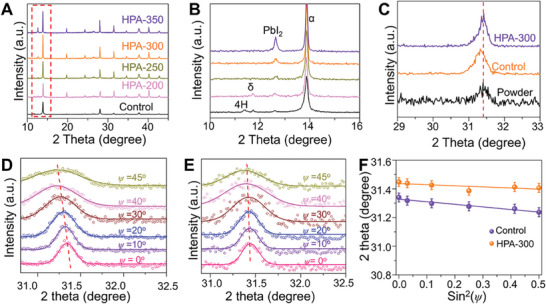
Lattice strain analysis of FAPbI_3_ Films. A) XRD patterns of control and HPA FAPbI_3_ treated FAPbI_3_ films at different temperatures. B) Enlarged view inside the red dashed box in Figure 3B. C) GIXRD spectra of powder FAPbI_3_ and control, HPA‐300 FAPbI_3_ film. D,E) GIXRD spectra with different instrument tilt angle values of control FAPbI_3_ film HPA‐300 FAPbI_3_ film. F) Lattice strain analysis by linear fitting of 2*θ*‐sin^2^ *ψ* for control and HPA‐300 FAPbI_3_ film.

In order to explore the effect of HPA recrystallization on the quality of α‐FAPbI_3_ perovskite. First, the phase transition process is operated at different temperatures. **Figure** **2**A shows the XRD patterns of FAPbI_3_ deposited on FTO substrates with different annealing conditions. The enlarged view of the red dashed box in Figure 2A (as shown in Figure 2B) shows that there is a small amount of 4H‐FAPbI_3_ and δ‐FAPbI_3_ in the control α‐FAPbI_3_ film. As the temperature reaches 200 °C, the 4H‐FAPbI_3_ disappears, and there is still a weak δ‐FAPbI_3_ peak in the HPA‐200 XRD pattern. When the temperature reaches 250 °C, the δ‐FAPbI_3_ completely disappears, accompanying the full formation of a phase‐pure α‐FAPbI_3_ film. We further proved this by Grazing‐incidence wide‐angle X‐ray scattering (GIWAXS) (Figure S10, Supporting Information) and in‐situ XRD measurement (Figure S11, Supporting Information). The temperature continues to rise to 300 °C, and as the grain grows, we can acquire stable phase‐pure α‐FAPbI_3_ film with a large gain size. When the HPA temperature reaches 350 °C, pinholes begin to appear in the FAPbI_3_ film (Figure S12, Supporting Information). It could be caused by the decomposition of FAPbI_3_ regarding the thermogravimetric analysis (TGA) curve when the temperature exceeds 320 °C in Figure S13 (Supporting Information). As it is well known, the residual δ‐FAPbI_3_ forces the cubic phase to undergo a reverse phase transition back to the hexagonal phase at room temperature,^[^ ^21^, ^22^ ^]^ so it is very important to grow phase‐pure α‐FAPbI_3_ for film stability. In addition, recent research shows that strain is another key factor for perovskite stability.^[^ ^23^, ^24^ ^]^ The strain‐induced alteration of electronic band structures can further change the carrier dynamics of perovskites, since the effective mass of charge carriers is assessed by the band curvature extracted from first‐principles calculations. Perovskite films with tensile strain have smaller ion migration activation energy both in the dark or under illumination, whereas compressive strain increases the activation energy under the same conditions.^[^ ^25^, ^26^ ^]^ Lattice strain can be determined by comparing the interplanar spacing with the unstrained value. Recrystallization is an effective method to relax the lattice strain of perovskite film.^[^ ^27^ ^]^ To explore the effect of recrystallization on lattice strain, grazing incident X‐ray diffraction (GIXRD) is performed to evaluate the lattice strain. The peak of (012) crystallographic plane is chosen for analysis due to its high diffraction angle and multiplicative factor, which can provide the most reliable structure symmetry information.^[^ ^28^ ^]^ As shown in Figure 2C, the (012) peak of the control FAPbI_3_ film shifts to a lower angle from 31.41° to 31.32° as compared to the HPA‐300 FAPbI_3_ film and strain‐relaxed powder FAPbI_3_. By contrast, the peak position of HPA‐300 FAPbI_3_ film is consistent with the powder FAPbI_3_, suggesting the HPA‐300 FAPbI_3_ film has a similar strain‐relaxation of the powder FAPbI_3_, the lattice tensile strain remains in the control FAPbI_3_ film and the tensile strain can be released by recrystallization for the HPA processed films. The classical 2*θ*‐sin^2^ *ψ* measurement is used to further reveal the lattice strain in FAPbI_3_ film, where *θ* and *ψ* represent the diffraction angle and the tilt angle, respectively.^[^ ^29^ ^]^ As shown in Figure 2D, by varying *ψ* from 0° to 45°, the diffraction peak is gradually shifted to a lower angle, indicating the control FAPbI_3_ film has lattice tensile strain in the in‐plane direction.^[^ ^26^, ^28^, ^29^ ^]^ In general, sin^2^ *ψ* and 2*θ* obey a linear relationship, the slope of the fitting line represents the magnitude of the residual strain. As depicted in Figure 2F, the control FAPbI_3_ film with tensile strain exhibits a negative slope, and the HPA‐300 FAPbI_3_ film has a smaller slope of absolute value suggesting that tensile strain can be released by high‐temperature recrystallization.

**Figure 3:**
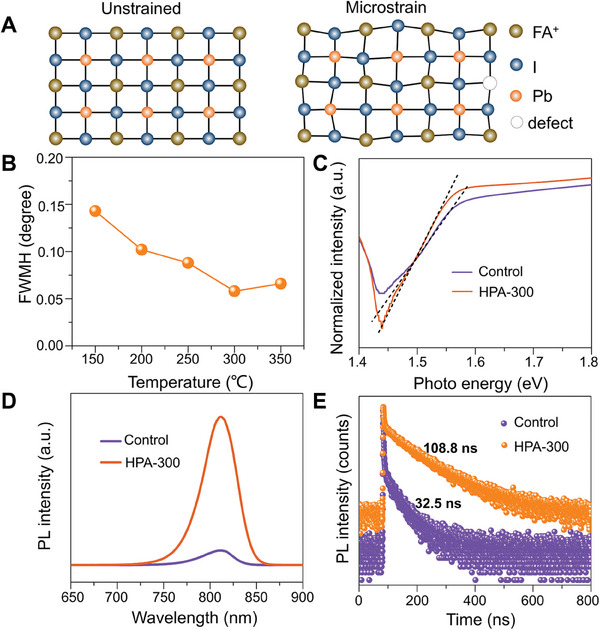
Micro‐strain analysis of FAPbI_3_ Films. A) Schematic diagram of microstrain. B) FWMH of the control and HPA‐treated FAPbI_3_ films at different temperatures. C) Urbach energy. D) photoluminescence spectra. E) Time‐resolved PL.

Microstrain is another factor that affects the stability of perovskites, and it refers to the internal strain between grains or subgrains caused by the uneven deformation of each grain or subgrain of an object.^[^ ^24^, ^30^ ^]^ In general, the microstrain is caused by atomic misfits, phase transitions, light/bias stimulation, and grain boundaries (as shown in **Figure** **3**A). Microstrain can be estimated by Equation (1), where *t_wh_* is grain size, *ε* is microstrain and *β* is FWHM of XRD, and constant *C* is a correction factor.^[^ ^31^, ^32^ ^]^

(1) $$\beta \mathrm{Cos}\theta =\frac{C}{t_{\mathrm{wh}}}+2\varepsilon \mathrm{Sin}\theta$$

The formula illustrates that the existence of microstrain is manifested as a large FWHM and a small grain size. In order to discover the influence of the recrystallization process on the microstrain of the FAPbI_3_ film, we calculate the FWHM of XRD of the film annealed at different temperatures. As shown in Figure 3B, the FWHM of the film decreases down to a minimum of 0.058° at 300 °C with increasing temperature. It is a substantial decrease in comparison with the control FAPbI_3_ film of 0.143°, which corresponds to the increase in grain size after recrystallization by HPA treatment in Figure 1C. The Urbach Energy is defined as *E*_U_ = *k_B_T*/*σ*(*T*), where *σ*(*T*) is the steepness factor, *k*_B_ is the Boltzmann constant, and *T* is the absolute temperature.^[^ ^33^ ^]^ A large *E*_U_ value is a strong indicator that the samples suffer from a cumulative effect of impurities, inherent structural disorders, and electron‐phonon interaction in the absorption processes.^[^ ^34^ ^]^ *E*_U_ can be calculated from UV–vis spectroscopy (Figure S14, Supporting Information) by Equation (2).

(2) $$\alpha =\alpha_{0}\;exp\left(E/E_{u}\right)$$

where *α* is the absorbance coefficient, *α_0_* is a constant, and *E* is the excitation energy. As shown in Figure 3C, by HPA treated at 300 °C, *E_u_* decreases from 31.8 meV (the control FAPbI_3_ film) to 21.6 meV. The Bandgap calculated from UV–vis spectroscopy of control and HPA‐300 is both ≈1.52 eV, which shows that hot‐press at high‐temperature annealing will not change the bandgap of α‐FAPbI_3_ (Figure S15, Supporting Information). In addition, strains are linked to a higher trap density which can be demonstrated by photoluminescence (PL) spectra and time‐resolved PL (TRPL).^[^ ^28^, ^35^ ^]^ Figure 3D–E shows that the HPA‐300 FAPbI_3_ film has an enhanced PL intensity in comparison with the control film, addressing HPA‐300 could effectively reduce the defect density of FAPbI_3_ film. It can be attributed to the undistorted grain formation and grain boundaries decrease in the recrystallization at the high temperature. Moreover, the lowered defects and trap density can extend the carrier lifetime, which is evidenced by TRPL and fitted by a bi‐exponential Equation (3)^[^ ^36^ ^]^:

(3) $$f\left(t\right)=\sum_{i}A_{i}\exp\left(-t/\tau_{i}\right)+B$$

where A_i_ is the decay amplitude, τ_i_ is the decay time, and B is a constant. The related parameters are summarized in Table S1 (Supporting Information). The average PL decay time (τ_ave_) is calculated by following Equation (4).^[^ ^36^ ^]^

(4) $$\tau_{\mathrm{ave}}=\frac{\sum A_{i}T_{i}^{2}}{\sum A_{i}T_{i}}\;$$

As shown in Figure 3E, The TRPL lifetime of the control and HPA‐300 FAPbI_3_ films are 32.5 and 108.8 ns, respectively, the HPA‐300 FAPbI_3_ film exhibits obviously enhanced TRPL lifetime than that of the control film, demonstrating that HPA process effectively reduces bulk defects by recrystallization process. The PL mapping of the HPA‐300 FAPbI_3_ film in Figure S16 (Supporting Information) exhibits much stronger and more uniform PL than the control film. The above results show that the HPA treatment effectively improves the quality of the FAPbI_3_ film by high‐temperature recrystallization resulting in strain release.

**Figure 4:**
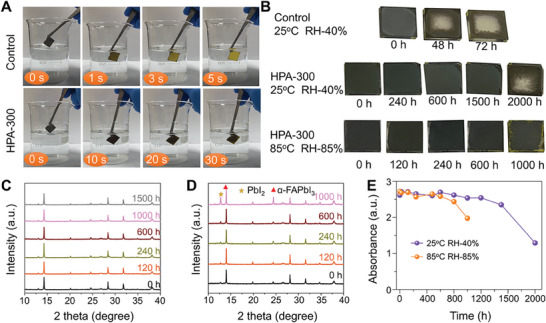
Improved damp‐heat stability of HPA‐treated FAPbI_3_ films. A) FAPbI_3_ films with conventional annealing method (control) and HPA treated dipped into deionized water at different time intervals. B) Photos of control and HPA‐300 FAPbI_3_ films exposed to damp‐heat conditions at different times. XRD patterns of HPA‐300 FAPbI_3_ film exposed to relative humidity (RH) of C) 40 ± 5% at 25 ± 2 °C and D) 85 ± 5% at 85 ± 2 °C for different times. E) Evolution of absorption of HPA‐300 FAPbI_3_ film at 550 nm.

In order to understand the effect of HPA process on the stability of FAPbI_3_ films, both of the control and HPA‐300 FAPbI_3_ films are dipped in water without any surface treatment. The control FAPbI_3_ film promptly turns yellow within 2 s due to its fast decomposition by water, whereas the HPA‐300 FAPbI_3_ film still remains black after 20 s and completely turns yellow after 60 s (**Figure** **4**A; Movie S1, Supporting Information). It proves that the HPA‐300 film has much better water resistance than the control film. In addition, the water contact angle test in Figure S17 (Supporting Information) also shows that HPA‐300 FAPbI_3_ film has better hydrophobicity than the control film. Water molecules prefer to react with the perovskite first at the grain boundaries, the HPA recrystallization process reduces defects at grain boundaries and releases the strain of perovskite film, thus exhibiting higher hydrophobicity than the control film.^[^ ^37^ ^]^ As for the perovskite stability, the damp‐heat test is necessary for as‐grown α‐FAPbI_3_ films. Figure 4B could be seen that the control FAPbI_3_ film exhibits significant degradation after 72 h exposed to relative humidity (RH) of 40 ± 5% at 25 ± 2 °C, whereas HPA‐300 FAPbI_3_ film remains black and does not show any conversion to δ‐FAPbI_3_ after 1500 h (Figure 4C). Even directly being exposure to RH of 85 ± 5% at 85 ± 2 °C for 600 h, the HPA‐300 FAPbI_3_ film still keeps no signs of degeneration though a certain peak of PbI_2_ appears until increasing to 1000 h. Figure 4E shows the absorbance variation (at 550 nm) of the HPA‐300 FAPbI_3_ films as a function of exposure time under different damp‐heat conditions, that HPA‐300 FAPbI_3_ films maintain good absorption after experiencing long‐time exposure in damp‐heat environment. The above test results address that the FAPbI_3_ film after high‐temperature recrystallization has excellent stability, which is attributed to the high quality of strain‐relaxed α‐FAPbI_3_ with larger grain size. Studies have shown that tensile strain can reduce ion migration activation energy and defect formation energy of perovskite films.^[^ ^38^ ^]^ Recrystallization through HPA treatment releases the tensile strain in the FAPbI_3_ films, effectively preventing ion migration and defect formation in the perovskite film, so the stability of the HPA‐300 film is greatly improved. In addition, high‐quality films with large grain sizes over 3 µm and low optical defects are also favorable for improved stability.

**Figure 5:**
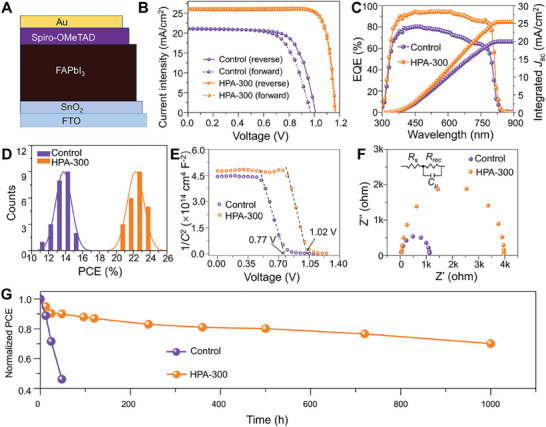
Photovoltaic performances. A) Schematic of the PSC with the structure FTO/SnO_2_/perovskite/spiro‐OMeTAD/MoO_3_/Ag. B) *J–V* curves of PSCs. C) EQE curves and integrated photocurrent. D) PCE statistical distribution of PSCs devices. E) Mott–Schottky plots. F) Nyquist plots of the PSCs. G) stability test of the PSCs.

High‐quality perovskite films are beneficial for the performance of efficient solar cells. To assess as‐grown α‐FAPbI_3_ film, solar cells based on FTO/SnO_2_/FAPbI_3_/Spiro‐MeOTAD/Au configuration are fabricated as shown in **Figure** **5**A (where FTO is fluorine‐doped SnO_2_ and Spiro‐MeOTAD is 2,2',7,7'‐tetrakis [*N, N*‐bis(methoxyphenyl)amino]c‐9,9'‐spirobifluorene). The device based on HPA‐300 FAPbI_3_ film achieves a champion PCE of 24.06% with negligible *J–V* hysteresis and an open‐circuit voltage (*V*_OC_) is 1.14 V, a short‐circuit current (*J*_SC_) of 25.42 mA cm^−2^, and a fill factor (FF) up to 83.07%. It is worth noting that the PCE is much higher than that based on the control FAPbI_3_ film, which is because the control FAPbI_3_ film shows much smaller grain sizes (e.g. Figure 1c–g), lower crystalline (e.g. Figure 1a), discontinuous film morphology (e. g. Figures S1a–S4a, Supporting Information), phase impurity (e.g. Figure 2b), poor absorption ability (e.g. Figure S11, Supporting Information), higher defect (e.g. Figure. 3c) and lower PL property (e.g. Figure 3d–e; Figures S7 and S8, Supporting Information). The detailed device parameters can be seen in Table S2 (Supporting Information). The typical external quantum efficiency of the devices in Figure 5C delivers 835 nm for the photo response edge of both the control and HPA devices. The distribution of the devices PCE measured from the PSCs based on control and HPA‐300 FAPbI_3_ is included in Figure 5D, which suggests a certain degree of reproducibility by the narrow distribution of PCE values. We have investigated the literatures on FAPbI_3_‐based perovskite solar cells in recent years to review their stability and efficiency, which are shown in Table S3 (Supporting Information). It can be seen from the Table that compared with other strategies and systems, the dopant and additive‐free α‐FAPbI_3_ perovskite solar cells prepared by the hot‐press‐recrystallization strategy show excellent stability in damp‐heat environment. To further study the device behavior of PSCs, Figure S18 (Supporting Information) shows the plots of the device PCE statistical distribution based on the FAPbI_3_ control films and HPA films at different temperatures. The results illustrate the devices based on HPA‐300 films have the best average PCE. Figure 5E shows Mott–Schottky plots of the control and HPA devices the flat‐band potential of the HPA device is higher than that of the control device, which is related to an increase in *V*_OC_.^[^ ^39^ ^]^ Electrochemical impedance spectra (EIS) are carried out to characterize the interfacial charge transport process in PSCs based on FAPbI_3_,^[^ ^40^ ^]^ the corresponding Nyquist plots and equivalent circuit model in Figure 5F. The interface resistance including the series resistance (*R*_S_) and the composite resistance (*R*_rec_) of the device can be obtained from the EIS curves. Compared with the control one, the higher *R*_rec_ value and lower *R*_S_ (Figure S19, Supporting Information) of HPA‐300 FAPbI_3_‐based PSC indicate a reduction in defects with the FAPbI_3_ film after high‐temperature recrystallization. Figure 5G presents the long‐term damp heat‐stability performance of the unencapsulated PSCs using thermally stable poly(triarylamine) as hole‐transporting material to rule out the degradation of the hole‐transporting layer. Unencapsulated devices are stored at 85 °C ± 2 °C in an oven under 85 ± 5% relative humidity under dark and ambient conditions, after 500 and 1000 h, the perovskite solar cell can still maintain ≈80% and 70% of the initial efficiency, while the control PCE decreases to ≈45% of initial PCE after 72 h. The improved stability of HPA device can be attributed to the high crystallinity, low defect density, and released strain of HPA FAPbI_3_ film.

## 3 Conclusion

In summary, HPA is a feasible method to grow phase‐pure, dopant‐free, stable α‐FAPbI_3_ film. The high‐temperature recrystallization and grain growth process can release the lattice strain and microstrain of the FAPbI_3_ film, resulting in higher stability, larger grain size, and less grain boundary of α‐FAPbI_3_ film than the control using conventional annealing methods. As a result, the corresponding PSCs have a high PCE and a stable behavior at 85 °C and 85% relative humidity. This work establishes a high‐temperature platform to achieve dopant‐free α‐FAPbI_3_ films, which is a new temperature‐induced solid‐phase secondary crystallization technology for perovskite films. Based on the stable and efficient performance of HPA α‐FAPbI_3_, which provides a reference idea for the large‐scale preparation of stable and efficient perovskite solar cells. Meanwhile, this solid‐state recrystallization technology provides a reference idea for realizing the all‐solid‐state preparation of perovskite films.

## Conflict of Interest

The authors declare no conflict of interest.

## Author Contributions

L.X. and X.X contributed equally to this work. All authors participated in the writing and revision of the paper.

## Supporting information



Supporting Information

Supplemental Movie 1

## Data Availability

The data that support the findings of this study are available from the corresponding author upon reasonable request.
