# Supplementary material for: High‐Temperature Driven Recrystallization for Stable Dopant‐Free α‐FAPbI3 Perovskite Solar Cells
Source: Adv Sci (Weinh). 2024 Nov 11;11(48):2408684. doi: 10.1002/advs.202408684 (PMC11672277; doi:10.1002/advs.202408684)
Supplement: Supplementary file 1 — Supporting Information [file ADVS-11-2408684-s002.docx]

**Supporting Information**

**High-temperature driven recrystallization for stable dopant-free α-FAPbI_3_ perovskite solar cells**

*Lingbo Xiao, Xiaoli Xu, Jie Zhao,* Chen Wang, Zheng Lu, Lutao Li, Liang He, Yu Chen, Guifu Zou****

L. Xiao, J. Zhao, C. Wang, Z. Lu, L. Li, G. Zou

College of Energy, Soochow Institute for Energy and Materials Innovations, Jiangsu Key Laboratory of Advanced Negative Carbon Technologies, and Key Laboratory of Advanced Carbon Materials and Wearable Energy Technologies of Jiangsu Province, Soochow University

Suzhou, 215000, China.

E-mail: jzhao@suda.edu.cn; zouguifu@suda.edu.cn

L. Xiao

Department of Physics, Zhejiang University of Science and Technology

Hangzhou, Zhejiang 310008, China

X. Xu

College of Materials and Chemistry, China Jiliang University

Hangzhou, 310018, China.

L. Li

Jiangsu Key Laboratory for Science and Applications of Molecular Ferroelectrics, Southeast University, Nanjing, 211189 P. R. China

L. He

National Photovoltaic Engineering Research Center, LDK Solar Co., Ltd

Xinyu, 338032, China.

Y. Chen

Beijing Synchrotron Radiation Facility, Institute of High Energy Physics

Beijing,100049, China

*E-mail addresses*: jzhao@suda.edu.cn (J. Zhao), zouguifu@suda.edu.cn (G. Zou)

**Experimental section**

**1 Materials**

Formamidinium iodide (FAI) was synthesized by reacting 10 g of formamidine acetate salt (≥99%, J&K Scientific) with 15 mL of hydroiodic acid (HI, ≥57 wt% in water, Aladdin) in a 100-mL round-bottom. The products were dissolved in ethanol, recrystallized from diethyl ether for four times, and finally dried at 60°C in a vacuum oven for 24 h. Lead iodide (PbI_2_)**_,_** (trifluoromethane)sulfonimide lithium salt (Li-TFSI), 4-tert-butyl-pyridine (tbp), chlorobenzene (CB) and isopropanol (IPA)，EuCl_3_⋅6H_2_O were purchased from Sigma-Aldrich. SnCl_2_•2H_2_O was purchased from Sinophrm Chemical Regent CO.，Ltd. Cobalt (III) FK209 bis(trifluoromethylsulfonyl)imide salt (FK209) was purchased from Greatcell Solar. Spiro-OMeTAD and Formamidinium lead triiodide (FAPbI_3_) powder were purchased from Advanced Election Technology CO., Ltd. N, N-dimethylformamide (DMF) and dimethyl sulfoxide (DMSO) were purchased from Alfa-Aesar. All materials were used as received without further modifications.

**2 Solar cell fabrication**

FTO glass is cleaned through ultrasonic cleaning by detergent, deionized water, acetone and ethyl alcohol for 10 min, respectively. After drying by dry-air blowing, it was treated by UVO for 15 min before use. Hole blocking layer SnO_2_ is deposited onto FTO substrate by chemical bath deposition method. 5 g urea was dissolved into 400 mL deionized water, followed by the addition of 100 μL mercaptoacetic acid and 5 mL HCl (37 wt%). Finally, 1.096 g SnCl_2_•2H_2_O and 0.88 g EuCl_3_⋅6H_2_O is dissolved in the above solution followed by stirring for 2 min (~ 0.012 M). The substrates are horizontally laid in a glass container filled with the diluted SnCl_2_•2H_2_O solution (~ 0.002 M) and heated to 70 °C in a lab oven for 3 h. Then it is washed by deionized water, dried by gas gun blowing, and followed by the annealing at 180°C for 1 hour. All the SnO_2_/FTO substrates were treated by UVO for 15 min. The perovskite precursor solution was prepared by mixing FAI (1.6M) and PbI_2_ (1.76M) in anhydrous DMF: DMSO 8:1 (V: V). The δ-FAPbI_3_ layer is fabricated by spin coating 35 μL perovskite precursor solution on top of the SnO_2_ layer by two consecutive spin-coating steps of 1000 rpm and 5000 rpm for 5 s and 15 s in a nitrogen glovebox, during the second step, 100 μL of chlorobenzene was poured on the spinning substrate 5 s prior to the end of the program. Then the films are preheated at 70°C to achieve the δ-FAPbI_3_ film. For the HPA process to prepare α-FAPbI_3_, the δ-FAPbI_3_ films are pressed for 10 min at the desired temperature (200°C, 250°C, 300°C or 350°C), then cool slowly to room temperature. For the preparation of control FAPbI_3_ films, the δ-FAPbI_3_ films directly annealed with a flat heater at 150°C for 20 min. After cooling down, the perovskite film is passivated by phenethylammonium iodide solution (2 mg/mL in isopropanol) with spin coated at 5000 rpm. After the fabrication of perovskite film, a hole-transport layer, Spiro-OMeTAD (72.3 mg mL^−1^ in chlorobenzene) solution with standard additives of 35 μL Li-TFSI (260 mg mL^−1^ in acetonitrile), 17 μL FK209 (165 mg mL^−1^ in acetonitrile) and 30 μL *t-*TBP was spin-coated on the perovskite film at 4500 rpm for 20 s. Finally, 80 nm of Au were deposited using thermal evaporation to complete the whole device.

**3 Characterization**

The surface morphology and cross-sectional images of perovskite films were captured using a scanning electron microscope (FEI Scios, American). The steady-state PL under an excitation at 520 nm and TRPL spectra were measured by Horiba fluorescence spectrophotometer (Fluoromax-4, France). The UV-vis absorbance spectra were measured on an UV-vis spectrophotometer (Lambda 750S, USA). The Mott-Schottky plots at a frequency of 1000 Hz and Nyquist plots at a bias voltage of 5 mV were recorded through an electrochemical workstation (CHI660E, China). X-ray diffraction (XRD) patterns were collected using Cu Kα radiation (Rigaku Smart Lab SE, 40 kV/40 mA). GIXRD patterns were recorded at an incident angle of 0.5°. The photocurrent density-voltage (*J*-*V*) curves of the PSCs were monitored using a Keithley Model (2400 SourceMeter, USA) under AM 1.5 solar simulator irradiation of 100 mW cm^-2^ (XES-300M2, Japan). The EQE spectra of the PSCs were measured using quantum efficiency measurement system (QE-R3018, Taiwan, China).

**4 Stability test**

Damp-heat stability of the FAPbI_3_ film was tested by exposing naked FAPbI_3_ film without further treatment under certain temperature, humidity conditions and room light. Absorbance and XRD of the films were recorded at intervals time. For the film stability test in water, naked FAPbI_3_ film without further treatment dipped in deionized water of 25°C. For the devices, ex-situ test was conducted by storing the devices in airtight box (85°C and RH~85%) with room light.


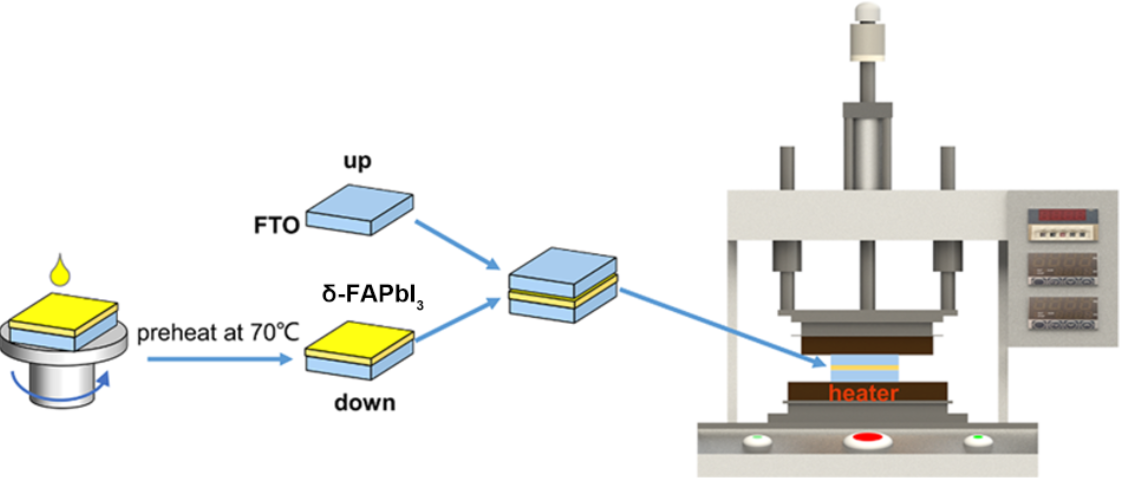


**Figure S1** Schematic diagram of HPA process. The as prepared films prepared by spin coating was quickly transferred to a flat heating table and kept at 70℃ for 30s to achieve the δ-FAPbI_3_ film, then the δ-FAPbI_3_ film placed in a hot press machine and covered with another FTO of the same size, kept at the desired temperature for 10min, after cooling, it is transfer to the glove box for the next step. (The entire HPA process is carried out in air)


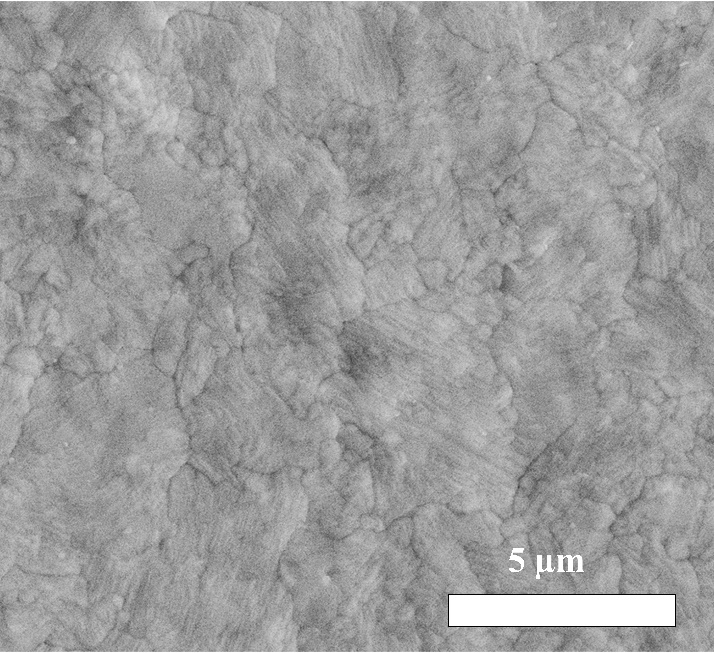


**Figure S2** Enlarged SEM view of Control film in Figure 1C


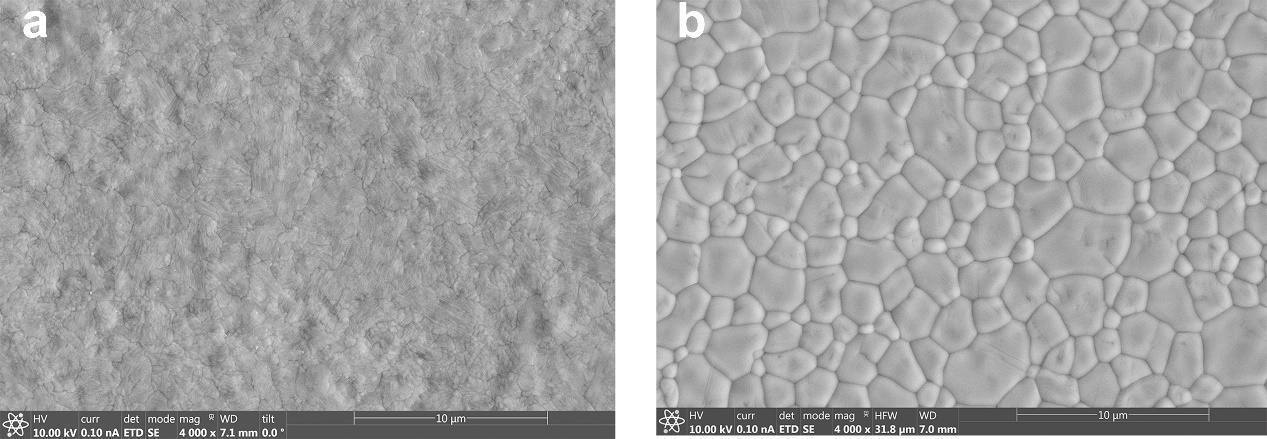


**Figure S3** Original SEM images of the control and HPA-300 FAPbI_3_ film. (a) control (b) HPA-300.


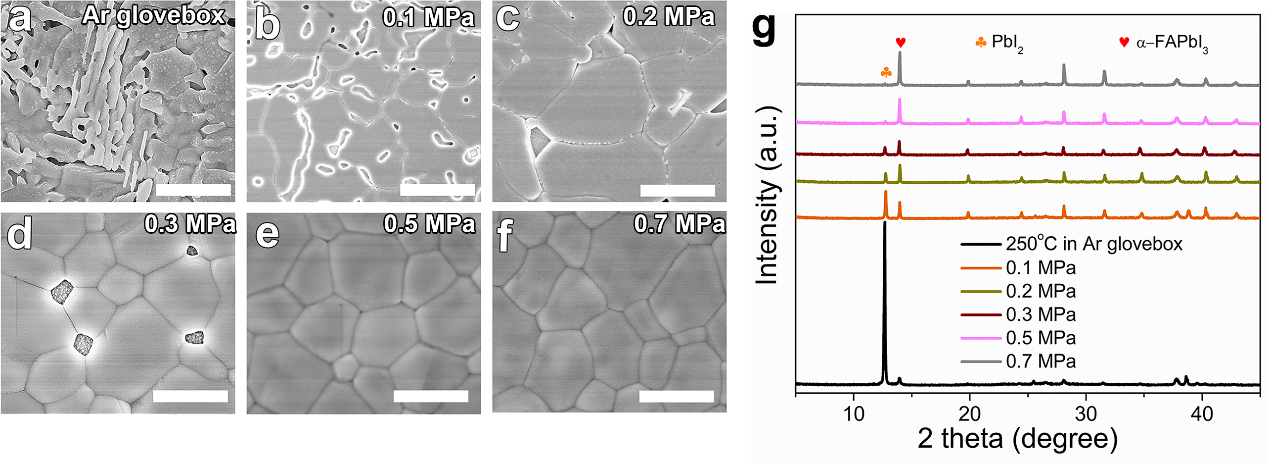


**Figure S4** The effect of pressure on FAPbI_3_ films. (a) The top-view SEM images of FAPbI_3_ films annealing at 250℃ in glovebox filled with argon. Top-view SEM image of FAPbI_3_ films by HPA annealing at 300℃ under (b) 0.1 MPa (c) 0.2 MPa (d) 0.3 MPa (e) 0.5 MPa (f) 0.7 MPa. (g) XRD spectra of FAPbI_3_ films by HPA annealing under different pressure. All annealing times are 10 min, and the scale in the figures is 3 µm.


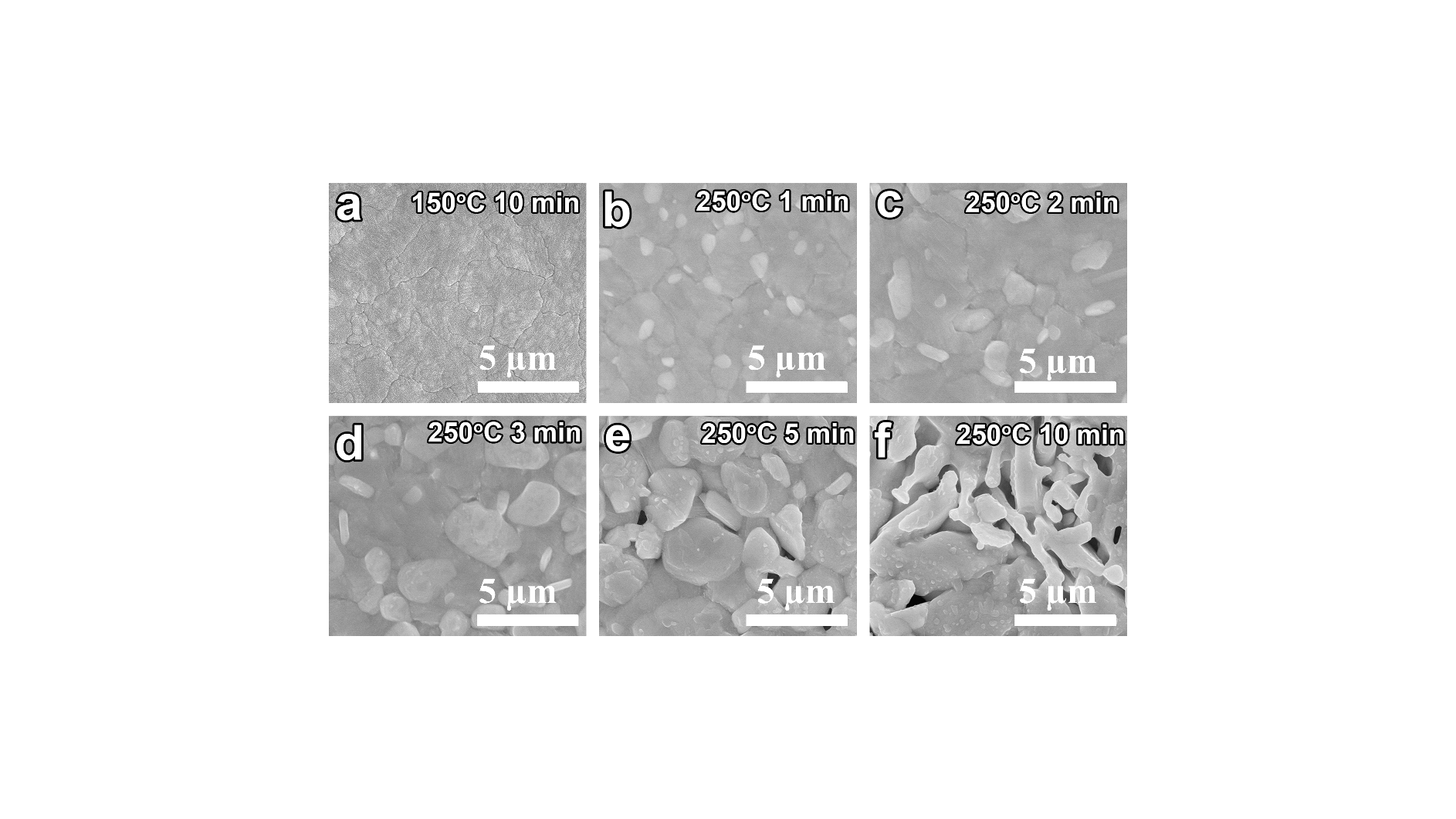


Figure. S5 Top-view SEM of FAPbI_3_ film prepared by conventional annealing (a)150℃ for 10 min. (b) 250℃ for 1 min (c) 250℃ for 2 min (d) 250℃ for 3 min (e) 250℃ for 5 min (f) 250℃ for 10 min.


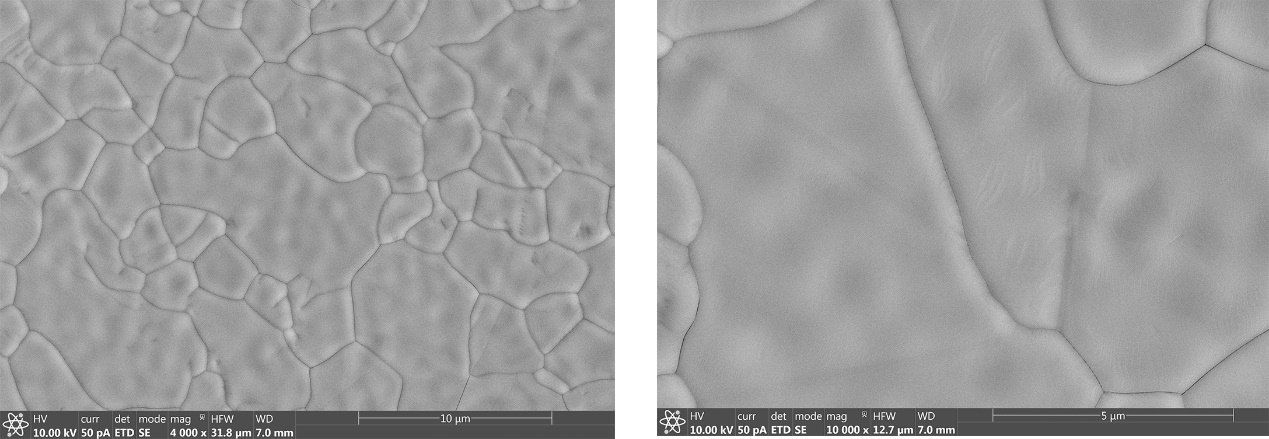


**Figure S6** Top-view SEM image of HPA-300 FAPbI_3_ film with large grain.


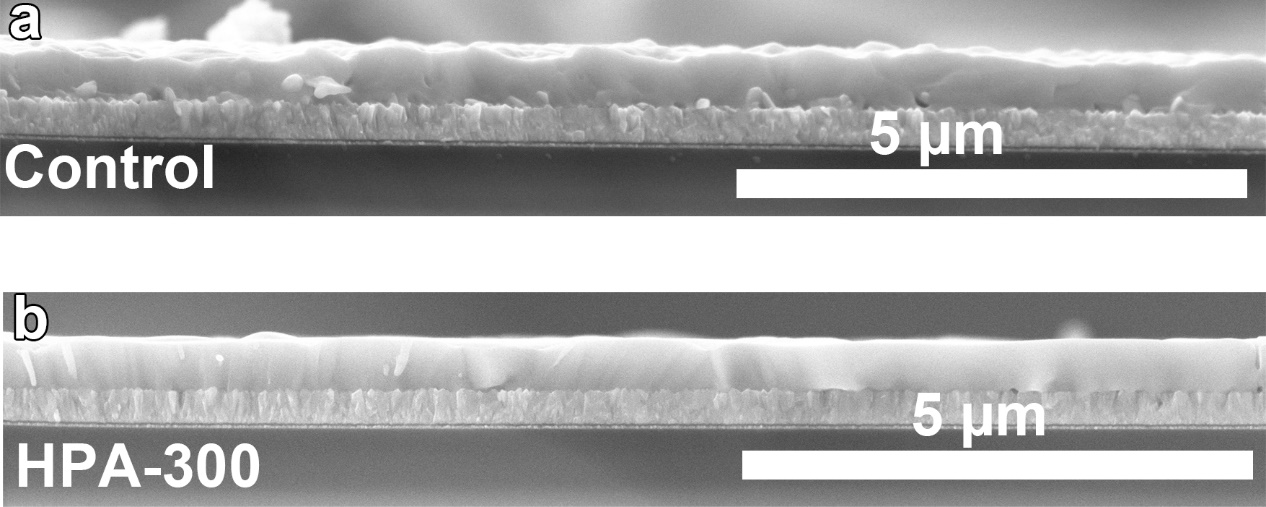


**Figure S7** Cross-sectional SEM images of control FAPbI_3_ film and HPA-300 FAPbI_3_ film (a) control (b) HPA-300.


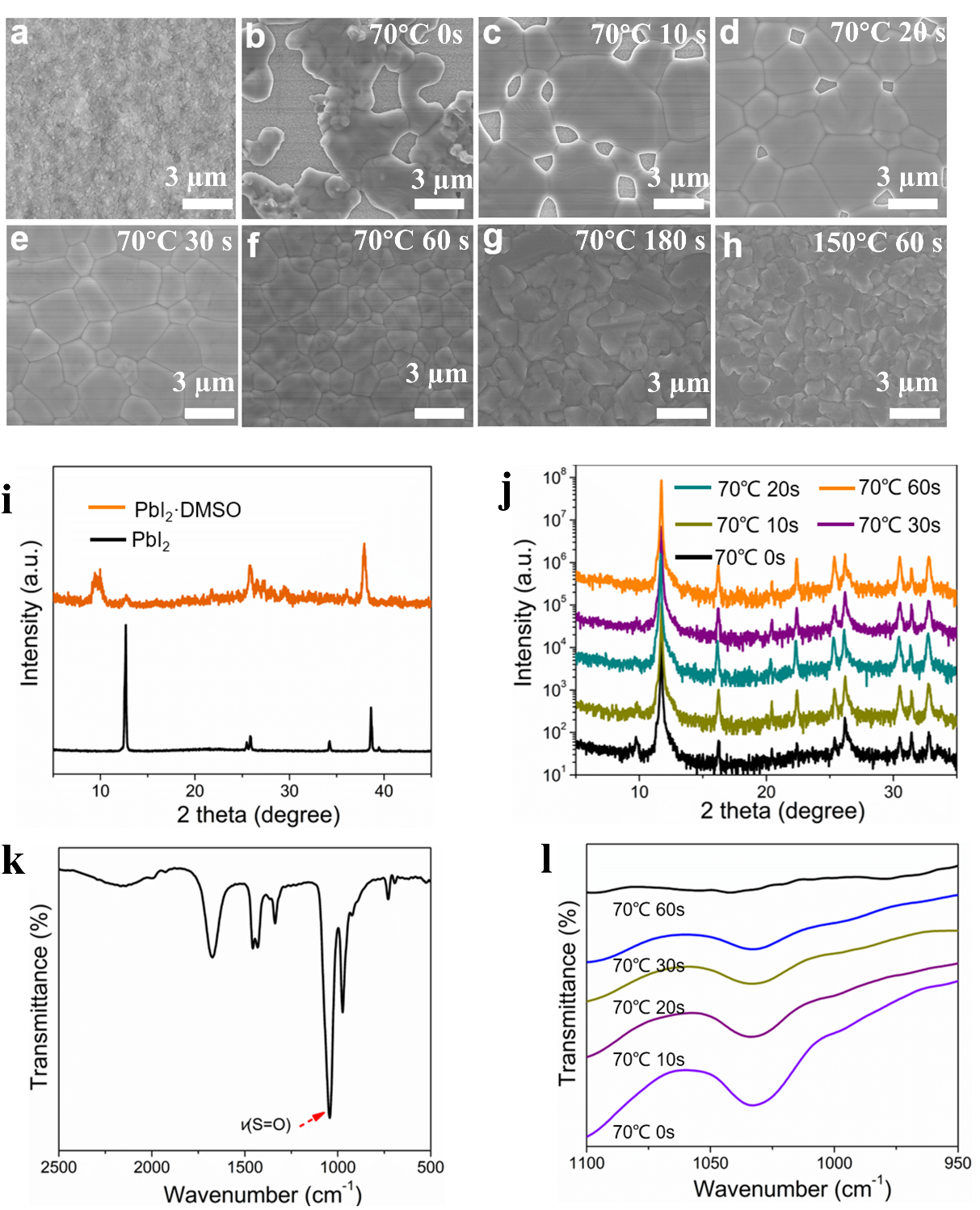


**Figure S8** Effect of preheating time on perovskite films. (a) Top-view SEM image of δ-FAPbI_3_ film preheated at 70°C for 60 s (b-h) Top-view SEM images of FAPbI_3_ films by HPA with different preheating times. (i) XRD spectra of PbI_2_ and PbI_2_·DMSO (j) XRD spectra of δ-FAPbI_3_ preheated at different times. (k) FTIR spectrum of DMSO (l) FTIR of spectrum δ-FAPbI_3_ preheated at different times.


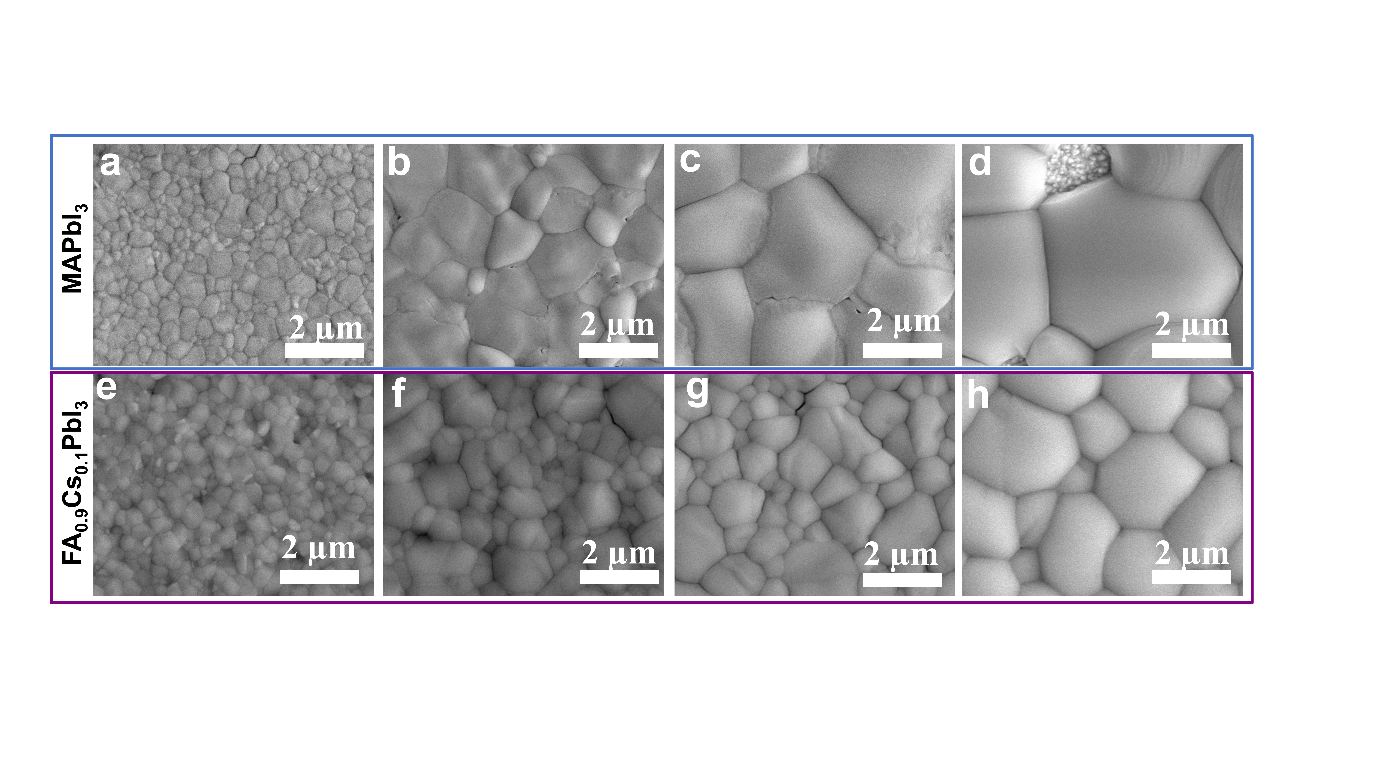


**Figure S9** The effect of HPA annealing under different temperature on the morphology of different components perovskite film. The top-view SEM images of MAPbI_3_ (a) traditional annealing at 100℃ (b) HPA annealing at 200℃ (c) HPA annealing at 250℃ (d) HPA annealing at 300℃. The top-view SEM images of FA_0.9_Cs_0.1_PbI_3_ (e) traditional annealing at 150℃ (f) HPA annealing at 200℃(g) HPA annealing at 250℃(h) HPA annealing at 300℃. All annealing times are 10 min.


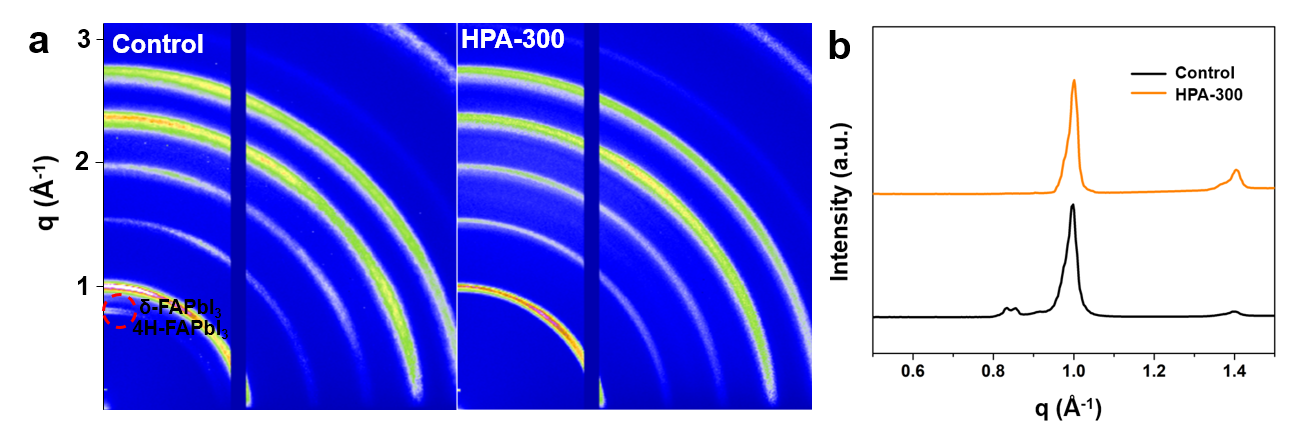


**Figure S10** (a) 2D GIWAX plots of FAPbI_3_ films (b) Radially integrated intensity of GIWAXS data.


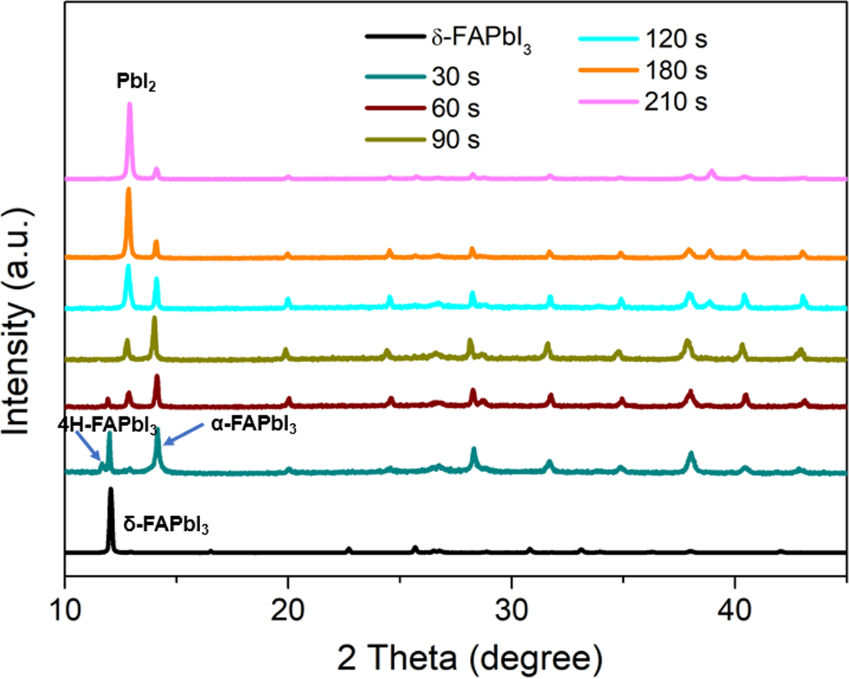


**Figure S11** In-situ XRD pattern of FAPbI_3_ annealed at 300℃


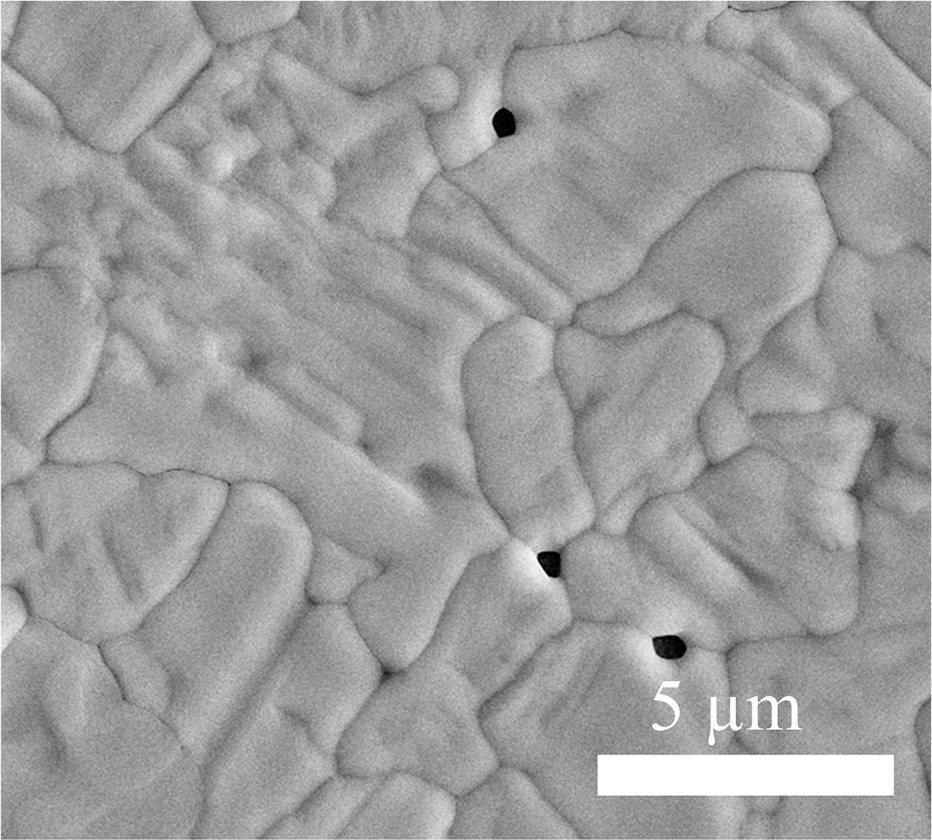


**Figure S12** SEM image of HPA-350 FAPbI_3_ film.


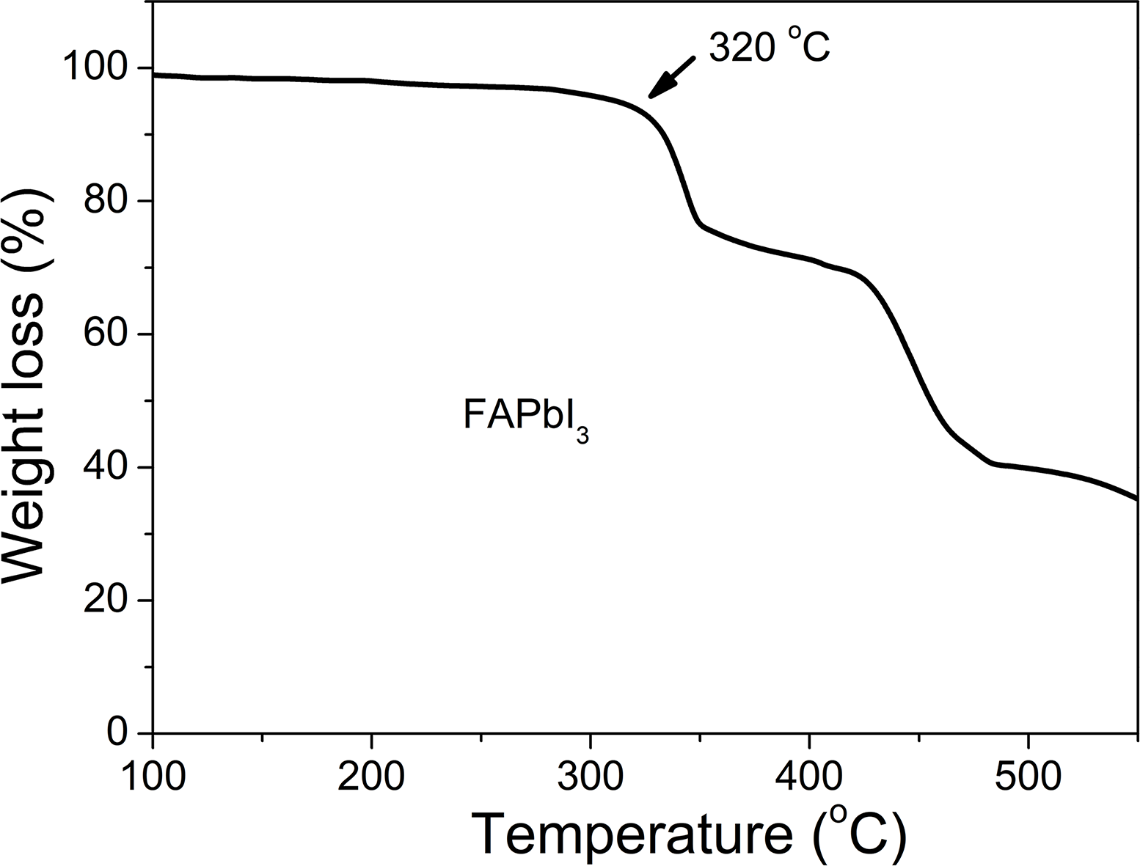


**Figure S13** TGA curve of FAPbI_3_.


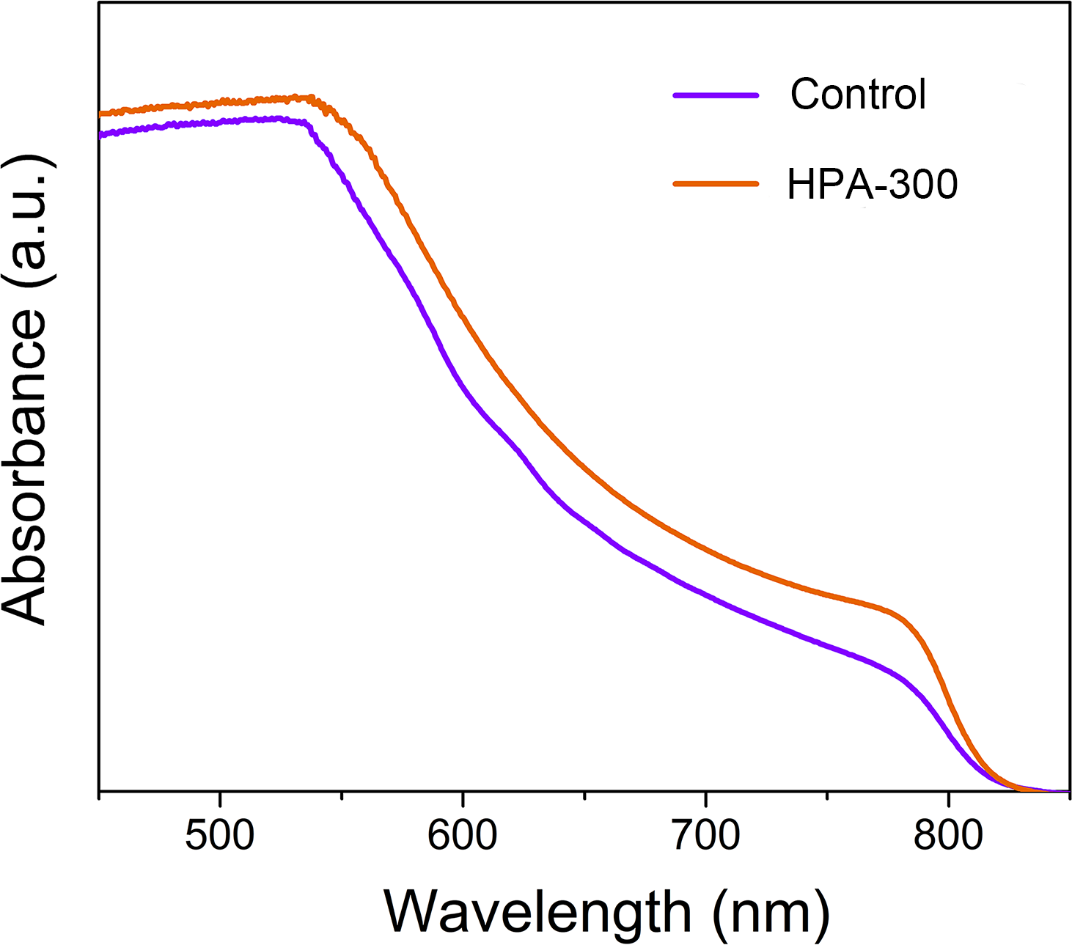


**Figure S14** UV-Vis spectroscopy of control FAPbI_3_ film and HPA-300 FAPbI_3_ film.


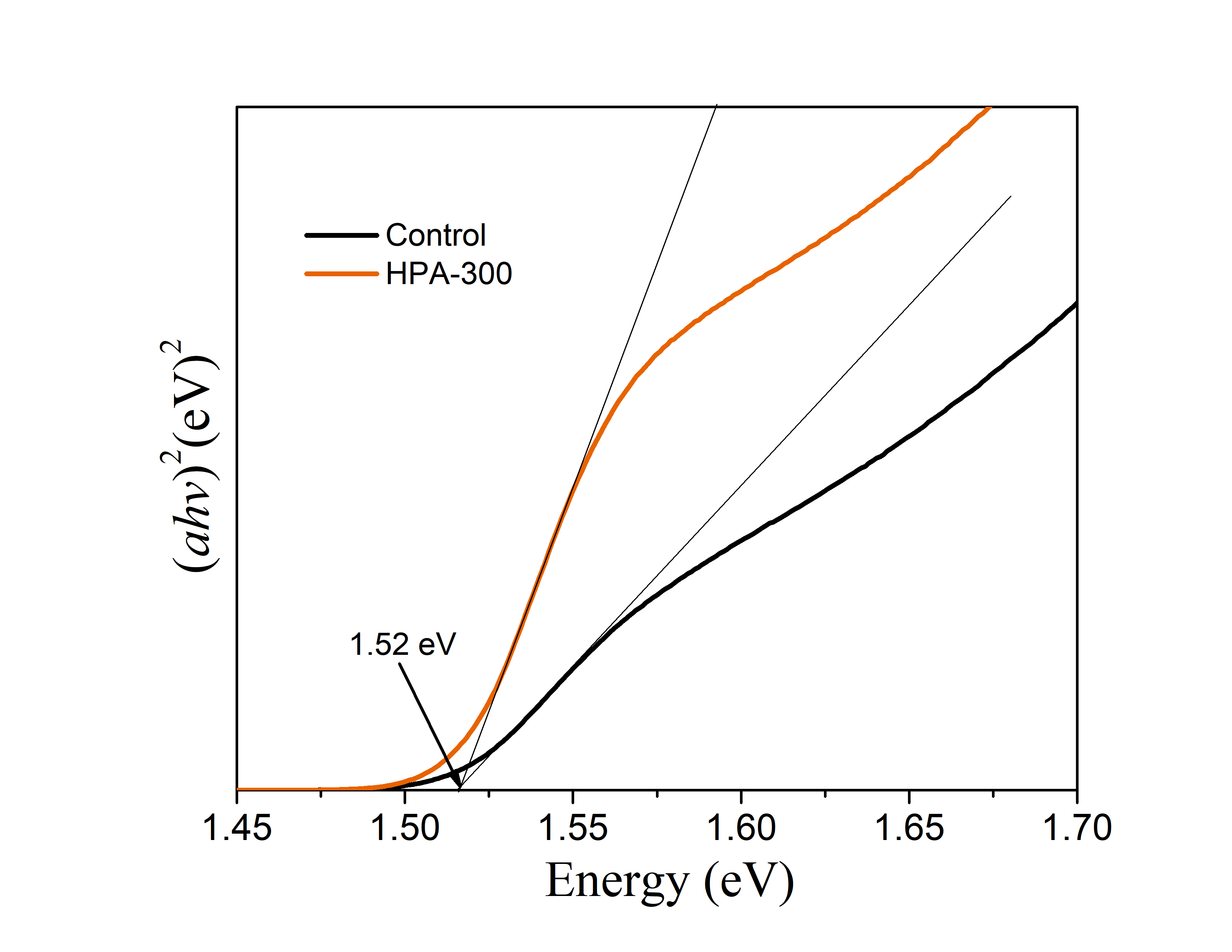


**Figure S15** Band gap calculated from UV-Vis spectroscopy


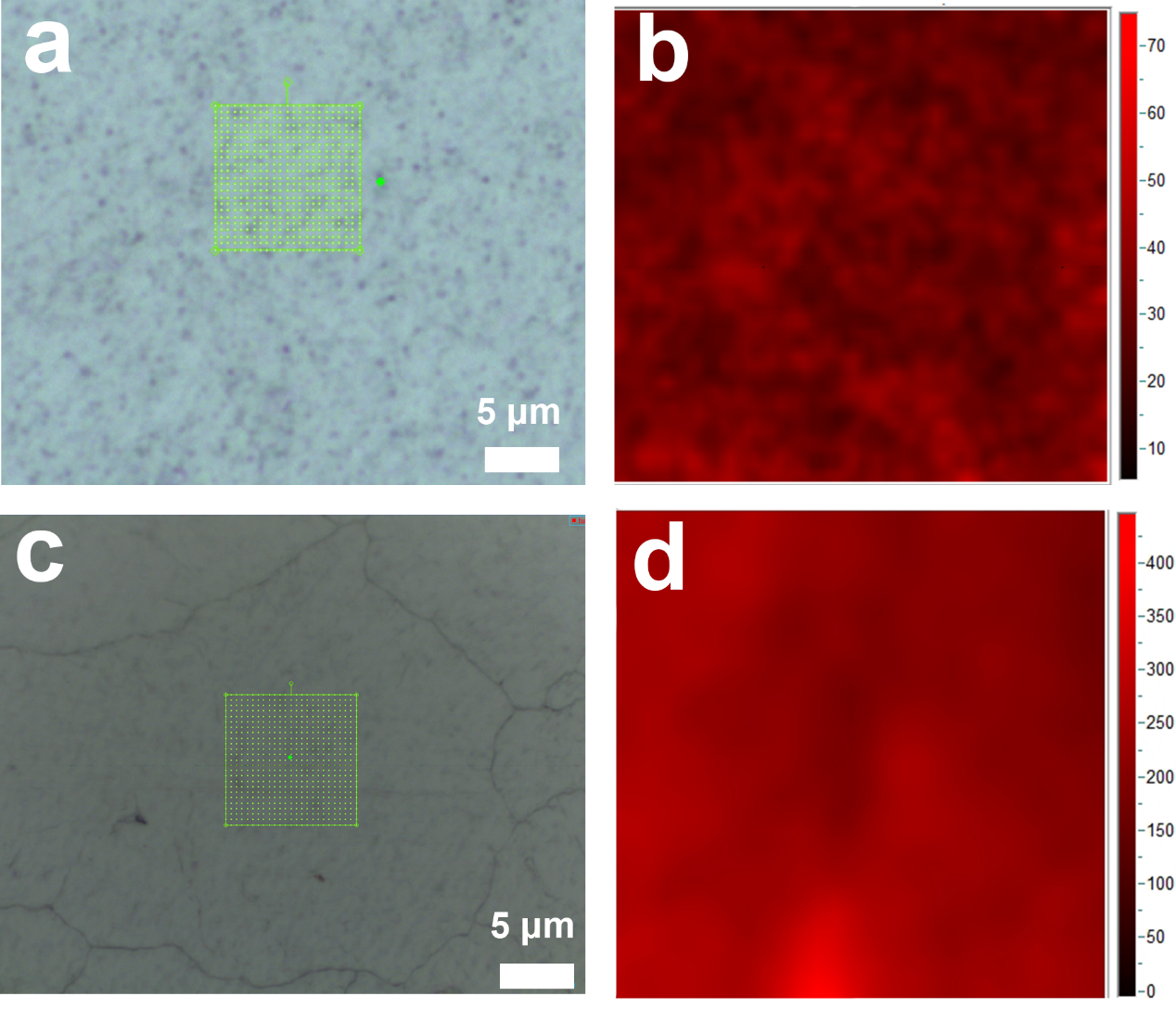


**Figure S16** PL mapping of (a-b) the control and (c-d) HPA-300 FAPbI_3_ film.


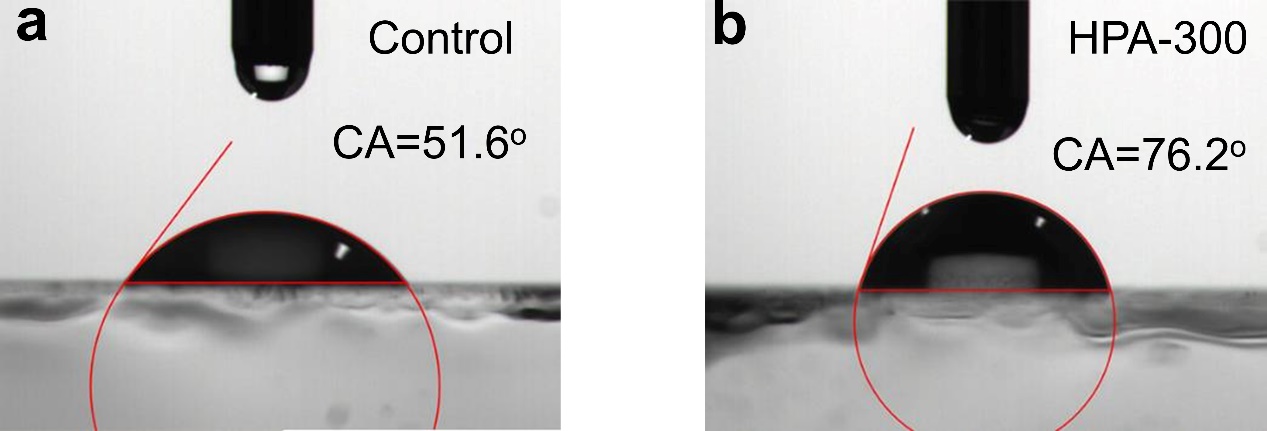


**Figure S17** Water contact angle test of (a) control film and (b) HPA-300 FAPbI_3_ film.


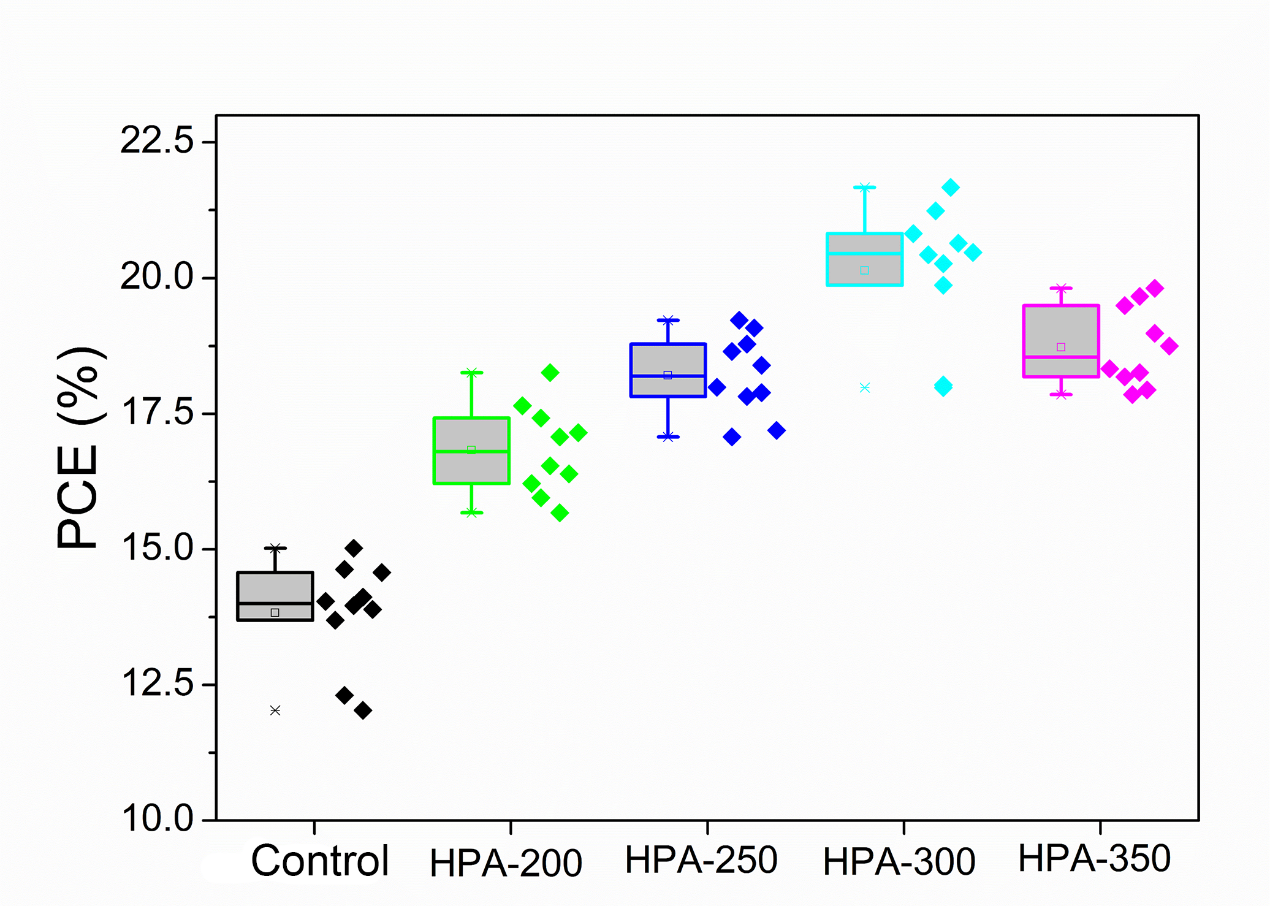


**Figure S18** PCE statistical distribution based on the FAPbI_3_ films of the control and HPA treated at different temperatures


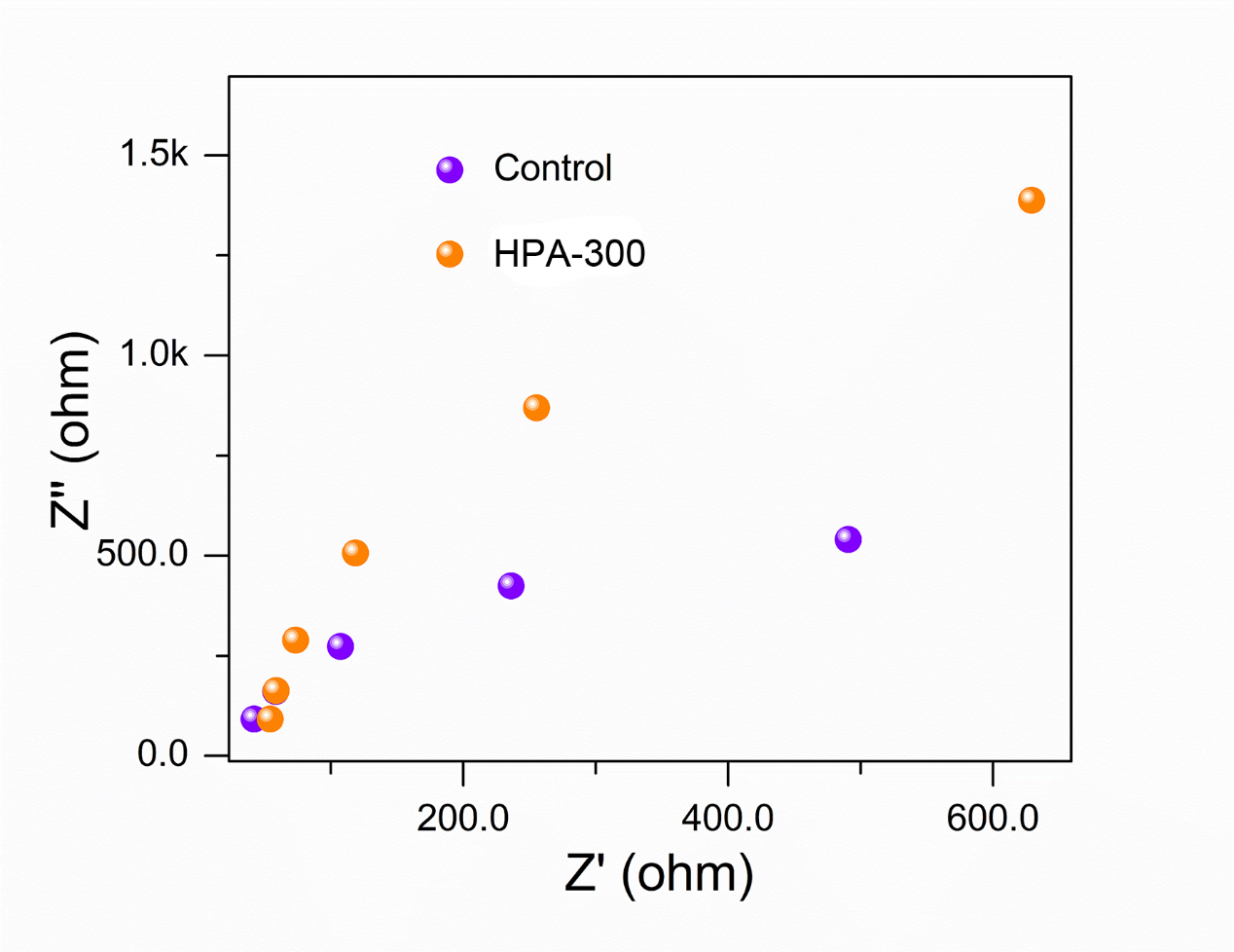


**Figure S19** Enlarge view of Nyquist plots of the PSCs based on the control and HPA-300 FAPbI_3_ films.

**Table S1** The fitting date from the TRPL spectra of FAPbI_3_ films

| Samples | B | A_1_ | t_1_(ns) | A_2_ | t_2_(ns) | τ_ave_(ns) |
| --- | --- | --- | --- | --- | --- | --- |
| Control | 0.00424 | 0.95063 | 1.57744 | 0.07655 | 52.29314 | 32.5 |
| HPA-300 | 0.01931 | 0.54944 | 2.66293 | 0.43543 | 112.02445 | 108.8 |

**Table S2**: Device parameters extracted from *J-V* curves of the best PSCs.

| Sample | Scan direction | *V*_OC_ (V) | *J*_SC_ (mA cm^-2^) | FF (%) | PCE (%) |
| --- | --- | --- | --- | --- | --- |
| Control | forward | 0.97 | 21.12 | 66.71 | 13.70 |
|  | reverse | 1.01 | 21.01 | 70.78 | 15.02 |
| HAP-300 | forward | 1.13 | 25.33 | 82.83 | 23.70 |
|  | reverse | 1.14 | 25.42 | 83.07 | 24.06 |

Table S3. PCE and stability of α-FAPbI_3_ based perovskite solar cells with recent literatures.

| PCE | Temperature and humidity | Time | Retained  initial efficiency ratio | Reference | Notes |
| --- | --- | --- | --- | --- | --- |
| 22.4% | 80℃, N_2_ | 300 h | 90% | *Adv. Energy Mater.* **2024**, 14, 2400932 | MA-free Cs-free, Solvent engineering |
| 21.6% | 25℃, N_2_ | 550 h | 90% | *Adv. Mater.*, 2022, 34, 2107850 | α-FAPbI_3_, aerosol-assisted crystallization |
| 24.16% | Ambient air | 1176 h | 95% | *Angew. Chem.* **2024**, e202403196 | Additive: MACl, N-aminoethylpiperazine  hydroiodide |
| 22.13% | Room temperature, 50±5% RH | 800 h | 92% | *Adv. Funct. Mater.* **2022**, *32*, 2200174 | Additive: MACl, Excess 5% mol PbI_2_ |
| 25% | Ambient atmosphere, AM 1.5G  illumination | 1000 h | 90% | *Energy Environ. Sci.*, **2024**, 17, 3375 | Encapsulated  Devices, Additive: MACl |
| 23.84% | 20-25℃,30-50% RH | 3000 h | 91% | *Adv. Energy Mater.,* **2023**, 13, 2300451 | single-crystal seeds crystallization |
| 25.6% | 60℃, 20% RH | 1000 h | 80% | *Nature*, **2021**, 592. 381 | Additive: MACl, FAHCOO |
| 23.24% | One-sun illumination | 800 h | 93% | *Joule*, **2023**, 7, 797-809 | Encapsulated, Zwitterionic AC^+^Cl^-^stabilized α-FAPbI_3_ |
| **24.04%** | **85^o^C, 85% RH** | **500 h** | **80%** | **This work** | **Hot-press at 300℃** |
|  |  | **1000 h** | **70%** |  |  |
